# Prognostic Abilities of Serial Neuron-Specific Enolase and Lactate and their Combination in Cardiac Arrest Survivors During Targeted Temperature Management

**DOI:** 10.3390/jcm9010159

**Published:** 2020-01-07

**Authors:** Seung Mok Ryoo, Youn-Jung Kim, Chang Hwan Sohn, Shin Ahn, Dong Woo Seo, Won Young Kim

**Affiliations:** Department of Emergency Medicine, Ulsan University College of Medicine, Asan Medical Center, 88, Olympic-ro 43-gil, Songpa-gu, Seoul 05505, Korea; chrisryoo@gmail.com (S.M.R.); yjkim.em@gmail.com (Y.-J.K.); schwan97@gmail.com (C.H.S.); ans1023@gmail.com (S.A.); leiseo@gmail.com (D.W.S.)

**Keywords:** cardiac arrest, lactic acid, neuron-specific enolase, prognosis

## Abstract

This study aimed to determine the prognostic ability of serial neuron-specific enolase (NSE) and lactate in cardiac arrest survivors treated with targeted temperature management (TTM) and to investigate whether a combination of NSE and lactate could increase prognostic information. This observational, retrospective, cohort study was conducted between January 2013 and December 2018; data were extracted from an out-of-hospital cardiac arrest registry. We collected serial serum NSE and lactate levels during TTM. The primary endpoint was poor neurological outcome at 28 days from cardiac arrest. Of all 160 included patients, 98 (61.3%) had poor neurological outcomes. Areas under the curves (AUCs) for NSE were 0.797, 0.871, and 0.843 at 24, 48, and 72 h, respectively (all *p* < 0.05). AUCs for lactate were 0.669, 0.578, 0.634, and 0.620 at 0, 24, 48, and 72 h, respectively (all *p* < 0.05). Although the combination of initial lactate and NSE at 48 h yielded the highest discovered AUC (0.877) it was not statistically different from that for the 48 h NSE alone (*p* = 0.692). During the TTM, NSE at 48 h from cardiac arrest was the most robust prognostic marker in comatose cardiac arrest survivors. However, a combination of the 48 h NSE with lactate did not increase the prognostic information.

## 1. Introduction

Prediction of neurological outcomes after cardiac arrest is critical since we have to distinguish late awakening from irreversible brain damage. Especially in the era of targeted temperature management (TTM), we should carefully assess the potential for improved neurological outcomes. However, there is no single test to reliably predict an outcome; a multimodal diagnostic approach is currently used to minimize prognostic uncertainty [1]. To predict a neurological outcome, various tests have been conducted in cardiac arrest survivors. Neurological examinations, imaging tests, neurophysiologic studies, and specific biomarkers such as neuron-specific enolase (NSE) or lactate have been used [2,3,4]. As biomarkers are relatively easily applied and are not affected by post-cardiac arrest care such as TTM, their importance has increased.

Current international guidelines recommend using NSE to assess neurological outcomes [5,6], as NSE is a marker of ischemic brain injury [7]. NSE is found in neuronal and neuroendocrine cells, and serum NSE concentrations correlate positively with the extent of anoxic–ischemic neurological injury and with malignant electroencephalography changes, after cardiac arrest [8,9,10]. Before the advent of TTM, a prospective study reported that a serum NSE concentration > 33 μg/L on days 1–3 after cardiac arrest was strongly associated with poor neurological outcome (false positive rate [FPR] 0%) [11]. However, results were more variable in subsequent studies, with unacceptably high FPRs being repeatedly reported [8]. Moreover, with TTM, the reliability of an NSE cut-off is unknown. Consequently, current guidelines do not recommend a specific cut-off value [12].

Lactate is produced from anaerobic metabolism after hypoxic tissue injury. During cardiac arrest, the anoxic state leads to lactate accumulation, and the serum lactate level is likely to be a useful marker of brain hypoxia, as also recommended in the Utstein guidelines [13]. Conversely, decreased lactate is a surrogate marker for adequate tissue perfusion after the return of spontaneous circulation (ROSC) [14]. Several studies addressed the association between lactate clearance and mortality and found that adequate clearance was associated with decreased mortality [15,16]. Most recently, decreased lactate over the first 12 h was associated not only with mortality but also with a good neurological outcome. However, area under the receiver operating characteristic curve (AUC) for predicting an adverse neurological outcome was still under 0.77 at 24 h [14].

Hence, this study was conducted for the patients receiving TTM treatment, to identify the best predictive time points for measuring serial NSE and lactate and the predictive value of NSE and lactate combined, as biomarkers for determining poor neurological outcomes.

## 2. Methods

### 2.1. Setting and Study Population

This registry-based, retrospective observational study was performed at an urban academic emergency department in the Republic of Korea. Data were extracted from the out-of-hospital cardiac arrest (OHCA) registry, which prospectively collected data for consecutive, comatose survivors of non-traumatic OHCA, who were treated with TTM between January 2013 and December 2018. The inclusion criteria of the registry were (1) age ≥18 years, (2) OHCA, (3) unconsciousness after ROSC, and (4) treated with TTM. The exclusion criteria of the registry were (1) intracranial hemorrhage, (2) acute stroke, (3) “do not attempt resuscitation” statement, (4) pre-arrest cerebral dysfunction, and (5) severe co-morbidity hence “expected to die within 180 days”. The study was approved by the Research Ethics Committee of the hospital (IRB No. 2015-1052). We divided patients into two groups—poor or good neurological outcomes at 28 days after cardiac arrest. The good neurological outcome was defined as cerebral performance category (CPC) grade 1 and 2. On the other hand, poor neurological outcome was defined as CPC grade 3 to 5.

### 2.2. TTM Protocol

In all comatose OHCA survivors, TTM was induced with intravenous cold saline and cooling devices (Blanketrol II, Cincinnati Subzero Products, Cincinnati, OH, USA); or Arctic Sun Energy Transfer Pad (Medivance Corporation, Louisville, CO, USA). The target temperature of 33 °C was maintained for 24 h; rewarming (0.25 °C/h to 37.0 °C) was then used to maintain normothermia until 72 h from ROSC. In patients treated with the normothermia protocol, we maintained body temperatures at 36.5–37.0 °C with the same cooling device until 72 h from ROSC. The temperature was monitored with an esophageal or rectal temperature probe. We used propofol, benzodiazepine, and opioids for sedation and analgesia. If required, a neuromuscular blocker was administered to control shivering. All patients received standard intensive care according to the institutional protocols.

### 2.3. Data Collection and Outcomes

Demographic and clinical data, including age, gender, previous medical history, initial vital signs, outcomes, and resuscitation profiles, such as arrest cause, initial rhythm, duration of resuscitation, emergency department management drugs, and shock were obtained. Laboratory values at admission were retrieved from the TTM registry. Initial and serial test results for NSE and lactate were obtained by the electronic medical record review. We obtained NSE data, 24, 48, and 72 h after ROSC, and NSE clearance was defined as 100× (24 h NSE–48 h [or 72 h] NSE) divided by the 24 h NSE (%). Serum lactate was measured at 0, 24, 48, and 72 h after ROSC, and lactate clearance was defined as 100× (initial lactate: 24 h (or 48 h or 72 h) lactate) divided by initial lactate (%). The primary endpoint was a poor neurological outcome at 28 days, which was defined as cerebral performance category 3–5 [17].

### 2.4. Statistical Analyses

Continuous variables were expressed as mean ± standard deviation or as median with interquartile range, if the assumption of a normal distribution was violated. Categorical variables were expressed as numbers and percentages. To analyze the baseline characteristics and laboratory examinations between the poor and good neurological outcome groups, the Student’s *t*-test was used to compare the means of normally distributed continuous variables; the Mann–Whitney U-test was used to compare the non-continuous variables. The Chi-squared or Fisher’s exact test was used to compare the categorical variables. We evaluated the prognostic value of serial NSE levels, NSE clearance, initial and serial lactate levels, and NSE and lactate clearance during TTM, and the prognostic value of NSE plus lactate, by area under the receiver operating characteristic curve (AUC).

Further, we analyzed logistic regression to find independent predictive factors for poor neurological outcome. Among potentially statistically significant risk factors (i.e., *p* < 0.1 in univariate analysis), we conducted multivariate analysis with a backward elimination method to find independently associated factors. We determined the odds ratio (OR) with a 95% confidence interval (CI) for each model. Finally, we tested the sensitivity, specificity, positive predictive value, and negative predictive value for the NSE plus lactate combination factor and found its cut-off value. All statistical tests were two-sided, and a *p* value < 0.05 was considered to be statistically significant. Statistical analyses were performed using SPSS for Windows, version 20.0 (SPSS Inc., Chicago, IL, USA), and MedCalc version 19.0.7 (MedCalc Software bvba, Ostend, Belgium).

## 3. Results and Discussion

**Figure 1:**
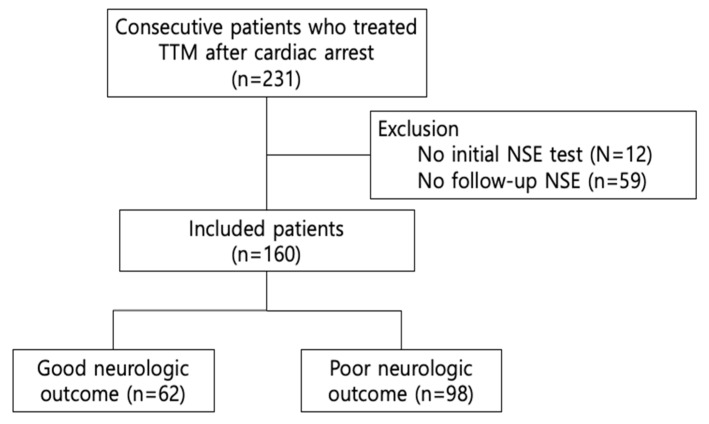
Flow chart of enrolled patients. NSE—neuron-specific enolase; TTM—targeted temperature management.

Of the 231 ‘patients treated with TTM after cardiac arrest’ that were extracted from the registry, we excluded 59 who had not repeated the NSE test and 12 without the initial NSE data. Finally, 160 patients were included—98 patients (61.3%) with a poor neurological outcome and 62 (38.8%) with a good neurological outcome (Figure 1).

Of all 231 enrolled TTM patients, we excluded patients without an initial or follow-up NSE test because we had to calculate the NSE clearance. Of the 160 patients included, 62 had a good neurological outcome, whereas the others had a poor neurological outcome.

The poor neurological outcome group was older (62.8 ± 15.9 vs. 52.6 ± 16.5 years, *p* < 0.05) and had more diabetes (31.6% vs. 16.1%, *p* < 0.05). However, witnessed cardiac arrest (69.4% vs. 85.5%, *p* < 0.05), presumed cardiac-origin arrest (41.8% vs. 75.8%, *p* < 0.05), and shockable initial rhythms (18.4% vs. 50.0%, *p* < 0.05) occurred less frequently in the poor versus good neurological outcome group. Hemoglobin levels (11.7 [9.8–14.0] vs. 14.1 [12.2–15.5], *p* < 0.05) and albumin levels (2.8 ± 0.6 vs. 3.4 ± 0.5, *p* < 0.05) were also lower in the poor neurological outcome group. Conversely, blood urea nitrogen (20.0 [14.0–31.5] vs. 15.0 [12.0–19.0], *p* < 0.05) and C-reactive protein (0.4 [0.1–2.1] vs. 0.1 [0.1–0.5], *p* < 0.05) were higher in the poor neurological outcome group (Table 1).

Serum NSE levels at 24, 48, and 72 h, and NSE clearance at 48 and 72 h were significantly associated with poor neurological outcomes, and respective AUCs that predicted a poor neurological outcome were 0.805, 0.860, 0.825, 0.731, and 0.674. Corresponding optimal cutoff values were 52.3, 82.5, and 76.0 ng/mL, and 19.6%, and 85.1%. Serum lactate levels at 0, 24, 48, and 72 h were also associated with a poor neurological outcome, and the respective AUC values were 0.669, 0.578, 0.634, and 0.620. Corresponding optimal cut-off values were 10.3, 2.5, 2.2, and 1.1 mmol/L.

**Figure 2:**
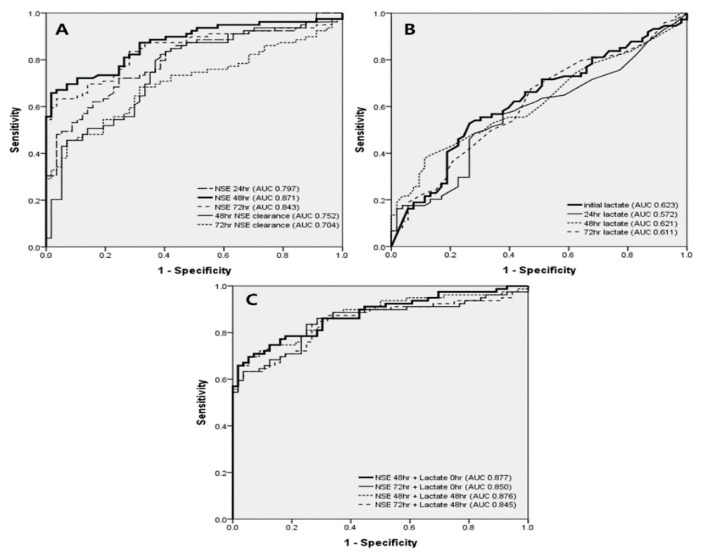
AUC values for serial NSE, lactate, and their combination. (**A**). AUC for serial NSE and NSE clearance—48 h NSE was the highest, followed by 72 h NSE. (**B**). AUC for serial lactate—initial lactate was the highest, followed by 48 h lactate. (**C**). AUC for combination of 48 h or 72 h NSE with initial or 48 h lactate—48 h NSE + initial lactate was the highest value. AUC—area under the receiver operating characteristic curve; NSE—neuron-specific enolase.

We combined NSE at 48 and 72 h with the initial lactate levels—AUCs were 0.882 for the 48 h NSE + initial lactate, and 0.840 for the 72 h NSE + initial lactate (Table 2; Figure 2). 

When we conducted multivariate logistic regression analysis (after adjustment for variables with *p* < 0.1 in univariate analysis), the 48 h NSE + initial lactate (OR 1.07; 95% CI: 1.02, 1.12; *p* < 0.05) and initial shockable rhythm (OR 0.01; 95% CI: 0.00, 0.32; *p* < 0.05) were independently associated with neurological outcome (Table 3).

For the 48 h NSE, the optimal cut-off level was 83 ng/mL, and sensitivity, specificity, positive predictive value, and negative predictive value for a poor neurological outcome were 67.4%, 98.3%, 98.5%, and 65.6%, respectively. Moreover, when 48 h NSE was 107 ng/mL, the FPR was 0%. 

The univariate analysis for the prediction of 28-day poor neurological outcomes associated with each optimal cut-off value was OR 121.8 (95% CI: 16.1, 920.5) for a 48 h NSE of 83 ng/ml; OR 3.1 (95% CI: 1.6, 6.2) for an initial lactate value of 10 mmol/L; and OR 127.8 (95% CI: 16.9, 966.8) for a 48 h NSE + an initial lactate value of 94. However, the AUC for the 48 h NSE + the initial lactate combination did not differ significantly (*p* = 0.692) from the value for 48 h NSE.

In this study, we found that the 48 h NSE had the most predictive value among each serial levels of NSE and lactate. Although the AUC value for the 48 h NSE + initial lactate combination was 0.877, and this was an independent predictor, it was not statistically different from the AUC value for the 48 h NSE alone.

NSE is the neuronal form of the cytoplasmic glycolytic enzyme enolase. It is mainly located in neurons and neuroendocrine cells and its biological half-life is 24 h [18,19]. Since Zandbergen et al. [11] reported that NSE > 33 µg/L at any time during post-cardiac arrest care was associated with a poor outcome (95% CI of FPR 0–3%), many studies about NSE have been conducted. In the era of pre-TTM, most studies showed that the serum NSE levels at 24–48 h from an event were the most valuable times for predicting outcomes [20]. However, after TTM was introduced, many studies have reported different results. For example, one recent study reported that a 48 h NSE was a better predictor than the NSE measured at 24 or 72 h—the corresponding AUC values were 0.87, 0.78, and 0.80, and the optimal cut-off values were 70.0, 68.1, and 56.0 µg/L [21]. Conversely, another retrospective study reported that the AUC for 72 h NSE (0.88) was a better predictor than the AUCs at 24 h (0.73) and 48 h (0.85) [8].

In our study, the AUC value for the 48 h NSE (0.87) was greater than the values for 24 h NSE (0.80) and the 72 h NSE (0.84); the respective optimal cut-off values were 82.5, 52.3, and 75.3 µg/L. It seems that the most predictive NSE level was that obtained at 48 h, and the cut-off value also increased with development of the TTM protocol. Although the AUC values for the NSE clearances were lower than the AUC values for serial NSE levels, NSE clearances at 48 and 72 h were also associated with poor neurological outcomes (AUCs for 48 h and 72 h NSE clearance were 0.752 and 0.704, respectively; *p* < 0.001 for both). Another study reported that ΔNSE, defined as 100 × (2nd NSE [at 69–77 h from the event] − 1st NSE [at 18–24 h from the event]) divided by 1st NSE (%), was associated with neurological outcomes [22]. In our study, conclusively, all serial serum NSE levels had higher AUC values than the AUC values for a 48 h or 72 h NSE clearance.

The prognostic value of serum lactate levels was studied in OHCA patients. A previous meta-analysis reviewed 22 articles with 6553 participants and, although the constituent studies were highly heterogeneous, lactate levels at 24, 48, and 72 h from admission were associated with poor neurological outcomes [13]. Like previous studies, our study also showed that all serial serum lactate levels from 0–72 h were associated with neurological outcomes. However, all AUC values were below 0.670.

Elevated serum lactate level was associated with various factors. First, the initial lactate level reflected a previous condition of cardiac arrest and duration of hypoperfusion. Next, the lactate levels after ROSC might be associated with reperfusion injury, systemic inflammatory responses, myocardial dysfunction, and microcirculatory dysfunction induced by post-cardiac arrest syndrome [14]. These effects might aggravate hypoxic brain injury and produce poor neurological outcomes. Moreover, high-dose adrenergic vasopressors can increase serum lactate by stimulating beta-adrenergic receptors [23]. Cerebral vasoconstriction due to such high-dose vasopressors might influence brain hypoperfusion.

However, conflicting data exist about the predictive ability of lactate clearance in OHCA survivors. One study showed that a marked lactate decrease at 12 h was associated with improved survival and good neurological outcomes, although the lactate change at 24 h was not significantly associated [14]. In another study, lactate clearance was not a surrogate marker of neurological outcome or in-hospital mortality [24]. In our study, although all lactate clearances at 24 h (OR 1.017; 95% CI: 1.004, 1.030), 48 h (OR 1.023; 95% CI: 1.007, 1.040), and 72 h (OR 1.018; 95% CI: 1.002, 1.034) were linked with an improved 28-day survival, we also could not show an association with neurological outcomes. Hypoperfusion might be more dangerous when it affects brain tissue rather than other vital organs. Conclusively, our data showed that serum lactate levels had higher AUC values than lactate clearance; the AUC values for lactate levels at 0 and 24 h were better than values at other time-points.

In this study, we hypothesized that the combination of NSE and lactate would be a better predictive factor of neurological outcomes in OHCA patients. As NSE reflects direct neuronal cell damage and lactate is a marker of tissue hypoxia, even in the brain, the combination might accurately predict a poor neurological outcome [18,23]. Thus, we combined the two most predictive factors of each NSEs and lactates, which were the 24 h NSE, 48 h NSE, the initial lactate, and the 24 h lactate. The combination with the highest AUC (0.877) was 48 h NSE plus initial lactate, which was an independent predictor. Although the optimal cut-off value was 95.0, when the value for the combination was 113.5, sensitivity, specificity, positive predictive value, and negative predictive value were 60.0%, 100.0%, 100.0%, and 61.2%, respectively. Thus, no patient had a good neurological outcome at 28 days after ROSC if the cut-off value for the combination was >113.5. However, this result was derived from the prognostic value of NSE. AUC values for the NSE + lactate combination and single 48 h NSE were not statistically significantly different (0.877 vs. 0.871; *p* = 0.692).

A recent paper reported that lactate level, and the need for vasopressors in the immediate post-arrest period, were associated with poor neurological outcomes [25]. Kim et al. [24] also reported that as serum lactate levels increase, neurological outcomes become progressively worse. In our study, all serial lactate levels were also significantly associated with poor neurological outcomes. However, because the AUCs were lower in our cohort than other studies, there was little synergism for the combination of lactate plus NSE.

This study has several limitations. First, it was a single-center, retrospective study. It was an observational study, and considerable data were missing. Further, because we did not have an initial NSE level, we could not assess initial NSE kinetics. However, as the 24 h NSE level was significantly lower than the 48 h and 72 h levels, and as the half-life of NSE was 24 h, we could expect the initial NSE level to be less than that at other times. Although we found that the combination of 48 h NSE and initial lactate was significantly higher in poor prognosis group, its OR and 95% CI was not high; therefore, validation is required in a large-population, interventional study, which might clarify the exact time-point of NSE for predicting a neurological outcome. Further studies will be needed to find other factors that could improve predictability by combining the use of 48 h NSE. 

## 4. Conclusions

NSE levels, at all time-points up to 72 h of TTM were a more robust prognostic marker than lactate or lactate clearance in comatose cardiac arrest survivors treated with TTM. The most valuable time-point for biomarker assessment were 48 h for NSE and 0 h for lactate levels. However, a combined assessment of 48 h NSE plus initial lactate did not provide more prognostic information than the evaluation of 48 h NSE alone.

## Figures and Tables

**Table 1:** Baseline characteristics and cardiac arrest profiles between the poor and good neurological outcome groups.

Characteristics	Total (*n* = 160)	Poor Neurological Outcome (*n* = 98)	Good Neurological Outcome (*n* = 62)	*p* Value
Age, years	58.8 ± 16.9	62.8 ± 15.9	52.6 ± 16.5	<0.05
Male	108 (67.5)	61 (62.2)	47 (75.8)	0.074
Past medical history				
Acute coronary syndrome	26 (16.3)	14 (14.3)	12 (19.4)	0.397
Arrhythmia	17 (10.6)	8 (8.2)	9 (14.5)	0.204
Hypertension	59 (36.9)	40 (40.8)	19 (30.6)	0.194
Diabetes	41 (25.6)	31 (31.6)	10 (16.1)	<0.05
Malignancy	14 (8.8)	11 (11.2)	3 (4.8)	0.164
Vital signs				
Systolic pressure, mmHg	137.8 ± 54.1	140.0 ± 54.6	134.1 ± 53.6	0.562
Diastolic pressure, mmHg	76.7 ± 28.7	73.0 ± 25.1	82.8 ± 33.4	0.071
Pulse rate, beats/min	112.6 ± 32.7	116.8 ± 33.6	105.5 ± 30.2	0.067
Laboratory findings, initial				
White blood cell, 10^3^/μL	12.4 [9.0–16.9]	12.1 [8.4–16.8]	13.0 [10.0–17.9]	0.355
Hemoglobin, g/dL	12.5 [10.7–14.6]	11.7 [9.8–14.0]	14.1 [12.2–15.5]	<0.05
BUN, mg/mL	17.0 [13.0–26.0]	20.0 [14.0–31.5]	15.0 [12.0–19.0]	<0.05
Creatinine, ng/dL	1.2 [0.9–1.7]	1.3 [0.9–1.9]	1.2 [0.9–1.4]	0.081
AST, mg/dL	114.0 [52.0–225.3]	114.0 [47.0–236.3]	114.0 [52.8–189.5]	0.603
ALT, mg/dL	76.5 [33.0–140.8]	79.5 [26.8–150.8]	74.5 [40.3–154.5]	0.517
Albumin, g/dL	3.1 ± 0.7	2.8 ± 0.6	3.4 ± 0.5	<0.05
C-reactive protein, mg/dL	0.2 [0.1–3.5]	0.4 [0.1–2.1]	0.1 [0.1–0.5]	<0.05
Witnessed	121 (75.6)	68 (69.4)	53 (85.5)	<0.05
Bystander CPR	117 (73.1)	68 (69.4)	49 (79.0)	0.180
Arrest cause				<0.05
Presumed cardiac cause	88 (55.0)	41 (41.8)	47 (75.8)	
Other medical cause	37 (23.1)	26 (26.5)	11(17.7)	
Hanging	12 (7.5)	10 (10.2)	2 (3.2)	
Asphyxia	16 (10.0)	16 (16.3)	0 (0.0)	
Others	7 (4.4)	5 (5.1)	2 (3.2)	
Prehospital initial rhythm				<0.05
Shockable	49 (30.6)	18 (18.4)	31 (50.0)	
Non-shockable	71 (44.4)	64 (65.3)	7 (11.3)	
Unknown	40 (25.0)	16 (16.3)	24 (38.7)	
TTM protocol				0.223
Hypothermia	145 (90.6)	91 (92.9)	54 (87.1)	
Normothermia	15 (9.4)	7 (7.1)	8 (12.9)	

Values are expressed as median [interquartile range], mean ± standard deviation, or number (%). ALT—alanine transaminase; AST—aspartate transaminase; BUN—blood urea nitrogen; CPR—cardiopulmonary resuscitation; TTM—targeted temperature management.

**Table 2:** Prognostic ability of serial neuron-specific enolase and lactic acid measurements for a 28-day poor neurological outcome.

Characteristics	Poor Neurological Outcome (*n* = 9 8)	Good Neurological Outcome (*n* = 62)	*p* Value	AUC
Neuron specific enolase (NSE)				
NSE 24 h, ng/mL	88.8 [46.7–193.5]	35.6 [26.6–51.8]	<0.05	0.797
NSE 48 h, ng/mL	158.5 [48.2–325.9]	29.1 [20.0–42.8]	<0.05	0.871
NSE 72 h, ng/mL	155.5 [45.6–340.0]	25.7 [19.8–44.8]	<0.05	0.843
NSE 48 h clearance, %	−42.0 [−123.5–11.2]	28.1 [17.5–42.0]	<0.05	0.752
NSE 72 h clearance, %	−38.6 [−200.4–26.3]	26.0 [12.0–50.1]	<0.05	0.704
Lactate				
Initial lactate, mmol/L	10.3 [7.1–13.5]	7.5 [4.1–10.2]	<0.05	0.623
Lactate 24 h, mmol/L	2.5 [1.3–4.2]	1.7 [1.3–3.4]	<0.05	0.572
Lactate 48 h, mmol/L	1.5 [0.9–2.7]	1.2 [0.9–1.9]	<0.05	0.621
Lactate 72 h, mmol/L	1.4 [1.0–2.0]	1.0 [0.9–1.6]	<0.05	0.611
Lactate 24 h clearance (%)	71.3 [44.9–84.4]	73.3 [55.8–83.4]	0.581	0.515
Lactate 48 h clearance (%)	80.0 [68.1–89.4]	83.8 [71.3–80.3]	0.295	0.546
Lactate 72 h clearance (%)	82.9 [75.6–88.9]	84.9 [78.0–89.7]	0.462	0.524
Combination NSE and lactate				
NSE 48 h + initial lactate	169.3 [63.1–340.9]	37.4 [29.3–52.7]	<0.05	0.877
NSE 72 h + initial lactate	165.3 [56.3–353.0]	36.7 [27.5–51.6]	<0.05	0.850
NSE 48 h + 48 h lactate	160.9 [52.5–326.7]	28.8 [21.0–41.2]	<0.05	0.876
NSE 72 h + 48 h lactate	156.8 [46.9–341.8]	26.5 [20.7–46.7]	<0.05	0.845

Values are expressed as median [interquartile range]. AUC—area under the receiver operating characteristic curve; NSE—neuron-specific enolase.

**Table 3:** Univariate and multivariate analyses of factors associated with poor neurological outcomes.

Characteristics	Poor Neurological Outcome (*n* = 98)	Good Neurological Outcome (*n* = 62)	Univariate Analysis OR (95% CI)	*p* Value	Multivariate Analysis OR (95% CI)	*p* Value
Age	62.8 ± 15.9	52.6 ± 16.5	OR 1.04 (95% CI: 1.02, 1.06)	<0.05		
Male	61 (62.2)	47 (75.8)	OR 0.53 (95% CI: 0.26, 1.07)	0.076		
Past medical history						
Diabetes	31 (31.6)	10 (16.1)	OR 2.41 (95% CI: 1.08, 5.35)	<0.05		
Initial vital signs						
Diastolic pressure, mmHg	73.0 ± 25.1	82.8 ± 33.4	OR 0.99 (95% CI: 0.98, 1.00)	0.076		
Pulse rate, beats/min	116.8 ± 33.6	105.5 ± 30.2	OR 1.01 (95% CI: 1.00, 1.02)	0.071		
Witnessed cardiac arrest	68 (69.4)	53 (85.5)	OR 0.39 (95% CI: 0.17, 0.88)	<0.05		
Presumed cardiac cause arrest	41 (41.8)	47 (75.8)	OR 0.23 (95% CI: 0.11, 0.47)	<0.05		
Initial shockable rhythm	18 (22.0)	31 (81.6)	OR 0.06 (95% CI: 0.02, 0.17)	<0.05	OR 0.01 (95% CI: 0.00, 0.32)	<0.05
Laboratory findings, initial						
Hemoglobin, mg/dL	11.7 [9.8–14.0]	14.1 [12.2–15.5]	OR 1.00 (95% CI: 0.99, 1.00)	0.667		
BUN, mg/mL	20.0 [14.0–31.5]	15.0 [12.0–19.0]	OR 1.04 (95% CI: 1.01, 1.06)	<0.05		
Creatinine, ng/dL	1.3 [0.9–1.9]	1.2 [0.9–1.4]	OR 1.29 (95% CI: 1.00, 1.66)	0.050		
Albumin	2.8 ± 0.6	3.4 ± 0.5	OR 0.13 (95% CI: 0.06, 0.28)	<0.05		
C-reactive protein	0.4 [0.1–2.1]	0.1 [0.1–0.5]	OR 1.24 (95% CI: 1.03, 1.50)	<0.05		
NSE 48 h + initial lactate	166.9 [59.0–340.2]	37.4 [29.3–52.7]	OR 1.04 (95% CI: 1.02, 1.06)	<0.05	OR 1.07 (95% CI: 1.02, 1.12)	<0.05

Values are expressed as median [interquartile range], mean ± standard deviation, or number (%). Multivariate analysis was adjusted with age, sex, diabetes history, initial diastolic blood pressure, initial pulse rate, witnessed, presumed cardiac cause arrest, initial shockable rhythm, initial laboratory findings of hemoglobin, BUN, creatinine, albumin, C-reactive protein, and the 48 h NSE level + initial lactate level, which were *p* < 0.1 in the initial statistical analysis. BUN—blood urea nitrogen; CI—confidence interval; NSE—neuron-specific enolase; and OR—odds ratio. Multivariate analysis was done by a logistic regression analysis through a backward elimination method.
